# The Influence of Evidence in Medical Practice

**Published:** 1916-03-04

**Authors:** 


### The Hospital

The Workers' Newspaper of Administrative Medicine and Institutional Life, Administration, National Insurance and Health.

No. 1551, Vol. LIX. SATURDAY, MARCH 4, 1916. PRICE ONE PENNY.

### THE INFLUENCE OF EVIDENCE IN MEDICAL PRACTICE.

The work of the medical practitioner has as its underlying basis the observation and interpretation of evidence. Perhaps, in the popular view, this statement may be deemed more appropriate to another profession rather than to that of medicine. Yet it is necessary to affirm its complete accuracy. Indeed, one may go further and maintain that even more in medicine than in law is the weighing of evidence the heart and soul of professional activity. For the lawyer attaches to the term a technical and restricted meaning, and in any given issue refuses to contemplate as evidence anything which is not comprehended in his definition. He may be quite right in regard to the aim which he pursues, but inevitably in such circumstances some part of the field is excluded from his gaze, and he is to this extent aided by artificial rules. The physician, on the contrary, neither suffers such limitation nor does he receive adventitious assistance. To him the whole field of testimony is open, and he is, indeed, compelled to enter it. What in law would be put aside as hearsay and irrelevant may be of high importance in medicine, and in any event the practitioner must needs weigh such testimony before rejecting it. Therefore, in a pre-eminent sense, it is correct to say that medical work is based upon the collection and estimate of evidence. In this respect particularly medicine asserts its inclusion in the scientific catalogue, and must not permit its banishment from this arena merely because many of its activities lack the precision of the occupations of the laboratory. Here, again, the judgment demanded of the physician has to surmount difficulties which are much greater than those presented to the laboratory worker. This latter may largely fix the conditions of his experiment, and may attach to these and to his end-result the attractive quality of exact measurement. He marks off his problem and limits the body of evidence which it is necessary for him to contemplate. But no such limitation is possible to the practitioner of medicine, who, far from infrequently, has to deal with elements which may not be defined by either weight or measure. Individuality, personality, idiosyncrasy—these are factors difficult to define or explain, but they are not less real on this account. It is here that to the physician the problem is often a perplexed one, and that the need for culture of the judgment in the weighing of evidence becomes so imperative. A life devoted to such work, therefore, cannot fail, to one who will use the opportunity, to be a discipline in the growth of a capacity wisely and impartially to judge and to weigh evidence. A certain degree of intuitive knowledge and rule-of-thumb expertness may, no doubt, come from the mere repetition of experiences, but the broad view, the judicial mind, and the ability to distinguish the accidental from the essential are achieved only when medicine is used as an instrument of self-culture and a school of mental discipline.

There are certain influences in the medical life which militate against the scientific weighing of evidence, and which, so far therefore, rob this life of part of its value as a subjective experience. One of these is the need for prompt decision and, what is involved in this, the pressure of time under which the evidence must be weighed. This partly arises from the necessities of the situation and partly from the impatience of the public. The medical practitioner often has to act, perhaps with a view to save life or to lessen suffering, before he has had time or opportunity to collect all the evidence bearing on the issue. His concern is not with some calm academic debate in which the arguments pro and con may be deliberately presented and nicely adjusted. At least this is not his concern in many of the practical issues of his daily work. Hence it follows almost necessarily that under such influences the disciplinary value of the calm judging of evidence is in danger of being pushed into the background. The occasion demands speed rather than a measured review of all the facts, and though speed carries risks there are still more dangers in delay. Such experiences are, of course, notorious, and no one will advocate that they be treated in other than the practical fashion which governs modern medical practice. But because at times haste is imposed and the circumstances compel action rather than a mental discussion, this is no reason for pursuing the same order under conditions quite different from those just stated. And our point for the moment is that it is this latter atmosphere which may be freely enjoyed in medical practice by whoever will take the trouble to avail himself of it. The widest possible field of testimony may be explored without the restriction of forensic limitations, and to the facts and statements thus collected may be applied the processes of review, consideration, and judgment.

Another influence which operates against the habit of the collection and study of all available evidence in medical practice is, as already stated, the impatience of the public. Remembering that health, and possibly life, are at stake, such impatience is not unnatural. The practitioner must promptly apply a name to the patient's illness, or otherwise he will be regarded as both an ignorant and an unsafe guide. To wait for the collection of more evidence is construed as a proof of weakness and fallibility. It is not possible altogether to resist this demand, and, indeed, it may be necessary at times to supply the patient with some definite phrase even as a therapeutic measure. Time and education and confidence born of previous experience will alone help the patient and his friends to realise that it may be quite possible to advise a sick man and to take full care of him, even though sufficient grounds for an exact diagnosis have not yet been collected. This and other difficulties and hindrances may be allowed, but they do not touch the contention that, properly used, the life of the medical practitioner is occupied by processes which involve the cultivation of the judicial faculty, and that out of this may be taken influences which make medicine not merely a craft having objective utility, but also a school possessing subjective values.

